# The interaction between antenatal care and abnormal temperature during delivery and its relationship with postpartum care: a prospective study of 1,538 women in semi-rural Uganda

**DOI:** 10.1186/s12884-022-05207-8

**Published:** 2022-11-21

**Authors:** Nicholas E. Rahim, Joseph Ngonzi, Adeline A. Boatin, Ingrid V. Bassett, Mark J. Siedner, Godfrey R. Mugyenyi, Lisa M. Bebell

**Affiliations:** 1 Medical Practice Evaluation Center, Massachusetts General Hospital, Boston, USA; 2 Department of Obstetrics and Gynaecology, Faculty of Medicine, Mbarara University of Science and Technology, Mbarara, Uganda; 3 Department of Obstetrics and Gynecology and Center for Global Health, Massachusetts General Hospital, Boston, USA; 4 Department of Medicine, Division of Infectious Diseases, Medical Practice Evaluation Center, Massachusetts General Hospital, Harvard Medical School, Boston, USA; 5 Department of Medicine, Division of Infectious Diseases, Medical Practice Evaluation Center, Center for Global Health, Massachusetts General Hospital, Harvard Medical School, Boston, USA; 6 Mbarara University of Science and Technology, Mbarara, Uganda; 7 Department of Medicine, Division of Infectious Diseases, Medical Practice Evaluation Center, Center for Global Health, Massachusetts General Hospital, Harvard Medical School, 55 Fruit St, GRJ-504, Boston, MA 02114 USA

**Keywords:** Antenatal care, Postnatal care, Pregnancy, Delivery, Postpartum, Fever, Hypothermia

## Abstract

**Background:**

Postnatal care (PNC) is an important tool for reducing maternal and neonatal morbidity and mortality. However, what predicts receipt and maintenance in PNC, particularly events during pregnancy and the peripartum period, is not well understood. We hypothesized that fever or hypothermia during delivery would engender greater health consciousness among those attending antenatal care, leading to greater PNC engagement after hospital discharge and our objective was to evaluate this relationship.

**Methods:**

Women were prospectively enrolled immediately postpartum at Mbarara Regional Referral Hospital (MRRH). We collected postpartum vital signs and surveyed women by telephone about PNC receipt, fever, and infection at two and six weeks postpartum. Our outcome of interest was receipt of PNC post-discharge, defined as whether a participant visited a health facility and/or was hospitalized in the postpartum period. Our explanatory variables were whether a participant was ever febrile (> 38.0˚C) or hypothermic (< 36.0˚C) during delivery stay and whether a participant attended at least 4 antenatal care (ANC) visits. We used logistic regressions to estimate the association between ANC and fever/hypothermia with PNC, including an interaction term between ANC and fever/hypothermia to determine whether there was a modifying relationship between variables on PNC. Regression models were adjusted for age, marital status, parity, HIV serostatus, Mbarara residency, and whether the participant was referred to MRRH,

**Results:**

Of the 1,541 women, 86 (5.6%) reported visiting a health facility and/or hospitalization and 186 (12.0%) had an abnormal temperature recorded during delivery stay. Of those who reported at least one visit, 59/86 (68.6%) delivered by cesarean, 37/86 (43.0%) reported post-discharge fever, and 44/86 (51.2%) reported post-discharge infection. Neither ANC attendance, abnormal temperature after delivery, nor their interaction term, were significantly associated with post-discharge PNC. The included covariates were not significantly associated with the outcome.

**Conclusions:**

While the overall proportion of women reporting post-discharge PNC was low, those who reported visiting a health facility and/or hospitalization had high proportions of post-discharge fever, post-discharge infection, and cesarean delivery, which suggests that these visits may have been related to problem-focused care. No significant associations between ANC and PNC were observed in this cohort. Further research assessing ANC quality and PNC visit focus is needed to ensure ANC and PNC are optimized to reduce morbidity and mortality.

## Background

While neonatal and maternal mortality rates have been trending downward since the turn of the twenty-first century, they remain high in many resource-limited settings like Uganda [1]. In 2015, the country reported 343 maternal and 19,000 neonatal deaths for every 100,000 live births [2]. In addition to high mortality rates, there are more than 100 women with acute morbidities for every maternal death [3]. An estimated 59.9% of Ugandan women who gave birth reported four or more antenatal care (ANC) visits, while only 22.5% reported at least one postnatal care (PNC) visit [4]. A better understanding of gaps in the cascade of care would help identify areas for intervention that could reduce morbidity and mortality in resource-limited settings.

In order to improve maternal and child outcomes, one gap in knowledge is factors that influence the utilization of PNC. At PNC visits, providers review topics important to ensuring the mother-baby dyad are thriving, including neonatal dietary habits, neonatal immunizations, and maternal mental well-being [5]. Furthermore, PNC services are critical in preventing, quickly diagnosing, and treating delivery complications [6]. Since 2013, the World Health Organization (WHO) has recommended that PNC begin within the first 24 h after birth and comprise at least three additional visits during the 6-week postpartum period, ideally at three days, between seven and fourteen days, and at six weeks postpartum [5]. A better understanding of factors influencing PNC utilization could improve maternal and child outcomes. Similar to PNC, ANC entails interventions and counseling, such as prescribing antenatal supplements and physical activity counseling, to promote healthy pregnancies and deliveries [7]. In resource-limited settings, maternal socioeconomic status, caesarean delivery, distance from healthcare facility, and maternal perceptions of local health facilities, and ANC visits are associated with PNC attendance [8–10]. Conversely, residing in a rural dwelling, including outside of Uganda's capital city of Kampala, was negatively associated with early PNC (first 24 h after birth) among Ugandan women [11].

However, there are few data on the relationship between ANC and PNC outside of the early-postpartum period and how delivery-related factors modify this relationship. Therefore, we sought to address this gap in knowledge by analyzing prospectively collected perinatal data from a cohort of women living in semi-rural Uganda. Borrowing from models of health behavior that suppose that adverse events can influence future behavior, we hypothesized that adverse experiences during labor and delivery, specifically incident fever or hypothermia with clinical suspicion for sepsis, would engender greater health consciousness among those with more ANC and lead to greater PNC engagement outside of the first 24 hours [12].

## Methods

### Study site

This analysis was conducted with data collected from a cohort of participants prospectively recruited from Mbarara Regional Referral Hospital (MRRH) to determine the incidence of and risk factors associated with postpartum infections [13]. MRRH is the largest referral hospital in southwestern Uganda, an area with a population of approximately five million people living in mostly rural, agrarian settings.

### Study population

The parent study screened every woman who presented to MRRH for delivery or within six weeks postpartum after delivery at MRRH or other facility for study enrollment between March and October in 2015. Participants who provided written, informed consent were followed by research nurses who measured participant vital signs approximately every eight hours starting immediately after delivery, including oral temperature [13]. Participants not previously tested for human immunodeficiency virus (HIV) or with self-reported HIV-negative serostatus were tested for HIV. To be enrolled, participants or their surrogate decision-maker had to provide written informed consent in either English or Runyankole (the local language) and be reachable by phone after discharge for follow-up. Those who could not speak English or Runyankole, declined consent, were incapacitated and next-of-kin declined surrogate consent were excluded. The study was conducted in accordance with the ethical standards laid down in the 1964 Declaration of Helsinki and its later amendments and was approved by the institutional ethics review boards at Mbarara University of Science and Technology (08/10–14), Partners Healthcare (2014P002725/MGH), and the Uganda National Council of Science and Technology (HS/1729). All methods were carried out in accordance with relevant guidelines and regulations. Participants with at least one febrile (> 38.0˚C) or hypothermic (< 36.0˚C) oral temperature recorded during delivery stay were considered to have had an abnormal temperature. Additional data was collected from these participants and a random sample of normothermic participants, including chart review and interviews. Interviews were conducted in-person at discharge and again by phone at 2 and 6 weeks postpartum. To achieve the original study objectives, 1,581 normothermic individuals of the original cohort of 4,231 participants were selected for interviews and chart review to ensure at least a 4:1 ratio of normothermic: febrile/hypothermic participants. The 4:1 ratio was based on power calculations performed for the parent study, to ensure sufficient power for the primary analysis and to ensure positivity in each subgroup in the primary analysis. All participants underwent temperature measurement beginning immediately after delivery using an ADC ADTEMP Hypothermia Digital Thermometer. Postpartum participants with at least one oral temperature measure during delivery stay at MRRH, available data on reported ANC attendance, and chart review, discharge interview, and either two- or six-week postpartum interview data were included in this secondary analysis. Those whose records were missing any of the above or did not deliver at MRRH were not considered for this secondary analysis. Participants included in the parent study who were admitted during the postpartum period but did present to MRRH for delivery were also excluded.

### Defining variables of interest

Our primary outcome was a binary variable indicating whether a participant-reported hospitalization or visit to a healthcare facility for care at any point between discharge and 6 weeks postpartum, measured using 2- and 6-week postpartum phone interviews. During these follow-up phone interviews, participants were also asked about whether they had a fever and whether a healthcare provider told them that they had an infection. Our exposure variables were adequate ANC attendance, defined as self-reported attendance ≥ 4 visits this pregnancy, and abnormal temperature, defined as one or more febrile or hypothermic temperature measurements while hospitalized for delivery of the baby. Abnormal temperature during delivery was selected as our adverse intrapartum exposure because fever and hypothermia are associated with adverse maternal and child outcomes. Fever has been associated with postpartum hemorrhage, retained placenta, and chorioamnionitis. Hypothermia has been associated with hypotension, respiratory depression, sepsis, and also postpartum hemorrhage [14–17]. The threshold number of four ANC visits was selected to align with the Ugandan government and WHO recommendations on antenatal care at the time participants were enrolled [7, 18].

### Statistical analysis

We compared cohort characteristics between the following four subgroups: (1) normothermic participants who had < 4 ANC visits, (2) normothermic participants who had ≥ 4 ANC visits, (3) participants with an abnormal temperature who had < 4 ANC visits, and (4) participants with an abnormal temperature who had ≥ 4 ANC visits. Secondly, we estimated the proportion of each subgroup who reported at least one instance of hospitalization or healthcare facility visit during the 6-week postpartum period, both separately and as a composite outcome. Differences in cohort characteristics and proportion estimates were discerned using chi-squared tests.

Following this, we conducted a series of multivariable logistic regressions for each explanatory variable to explore the separate associations of ANC and abnormal temperature during admission for delivery with postpartum contact with healthcare. Lastly, we estimated a multivariable logistic regression with both independent variables and an interaction term to determine if there is a modifying relationship between the ANC attendance and abnormal temperature postpartum. All regressions were adjusted for the following participant characteristics: (1) age, (2) marital status at time of delivery, (3) parity, (4) residence in Mbarara, (5) referral to MRRH for delivery, and (6) HIV serostatus. Covariates were selected a priori based upon their relationships with the exposure variable and outcome of interest [11, 19–21].

Sensitivity analyses were conducted in which the outcome of interest was disaggregated to examine postpartum hospitalization and healthcare facility visits separately. We also performed additional sensitivity analyses using separate logistic regression models with febrile and hypothermic temperature groups disaggregated, modeling temperature both as a continuous variable, and as a dichotomous variable. The febrile group model excluded hypothermic participants and visa versa. Data were entered into a Research Electronic Data Capture (REDCap) database and analyzed using Stata (version 16.1, StataCorp, College Station, TX) [22].

## Results

### Enrollment and demographics

Overall, 4,231 individuals were enrolled in the parent study (> 99% of those eligible). Of those, 1,538 (36.4%) met inclusion requirements for this secondary analysis. The median duration of hospital stay was 2 days (interquartile range [1–3]). The four participant subgroups included the normothermic group with < 4 ANC visits totaling 373 (24.3%), normothermic with ≥ 4 ANC visits totaling 979 (63.7%), abnormal temperature with < 4 ANC visits totaling 54 (3.5%), and abnormal temperature with ≥ 4 ANC visits totaling 132 (8.6%). Individual characteristics by group are presented in Table 1. Overall, 86 participants (5.6%) reported visiting a health facility and/or hospitalization during the 6-week postpartum period. Of these, 13 (15.1%) were HIV-infected, 44 (51.2%) reported having a post-discharge infection, 37 (43.0%) reported having a post-discharge fever, and 59 (68.6%) delivered by cesarean. These outcomes are not mutually exclusive. Mean overall age was 25.2 (standard deviation 5.5) years, 589 (38.3%) participants were primiparous, 179 (11.7%) were HIV-infected, and 769 (50.1%) delivered by cesarean. The four groups were significantly different in age (*P* < 0.01), marital status (*P* = 0.01), parity (*P* < 0.01), delivery by cesarean (*P* < 0.01), and referral to MRRH (*P* < 0.01).

**Table 1:** Sociodemographic and obstetric characteristics of participants

Characteristic	< 4 ANC visits and normal temperature (*n* = 373)	≥ 4 ANC visits and normal temperature (*n* = 979)	< 4 ANC visits and abnormal temperature (*n* = 54)	≥ 4 ANC visits and abnormal temperature (*n* = 132)	*P*-value
**Demographic and obstetric**					
*Age*					
19 years or younger	43 (12)	115 (12)	13 (24)	31 (23)	< 0.01
20—34 years	290 (79)	776 (80)	38 (70)	99 (75)	
Older than 34 years	36 (10)	79 (8)	3 (6)	3 (2)	
*Married*	337 (90)	929 (95)	47 (87)	123 (93)	0.01
*Singleton pregnancy*	362 (97)	946 (97)	52 (96)	127 (95)	0.53
*Parity*					
1, primiparous	112 (30)	375 (38)	26 (48)	76 (58)	< 0.01
2–4, multiparous	208 (56)	495 (51)	18 (33)	49 (37)	
> 4, grand multiparous	53 (14)	109 (11)	10 (19)	7 (5)	
**Antenatal diagnoses**					
*HIV positive*	43 (12)	115 (12)	3 (6)	18 (14)	0.48
*Urinary tract infection*	12 (3)	24 (2)	2 (4)	0 (0)	0.21
*Syphilis*	5 (1)	20 (2)	1 (2)	7 (5)	0.06
*Malaria*	31 (8)	100 (10)	3 (6)	13 (10)	0.54
**Intrapartum factors and outcomes**					
*Cesarean delivery*	155 (43)	467 (49)	42 (81)	105 (81)	< 0.01
*Referred to MRRH*	41 (11)	114 (12)	19 (35)	28 (21)	< 0.01

Results reported as n (%) and *P*-values are calculated using Chi squared tests

*Abbreviations*: *ANC* Antenatal care, *HIV* Human immunodeficiency virus, *MRRH* Mbarara Regional Referral Hospital

### Descriptive statistics of postpartum interactions with healthcare

**Fig. 1:**
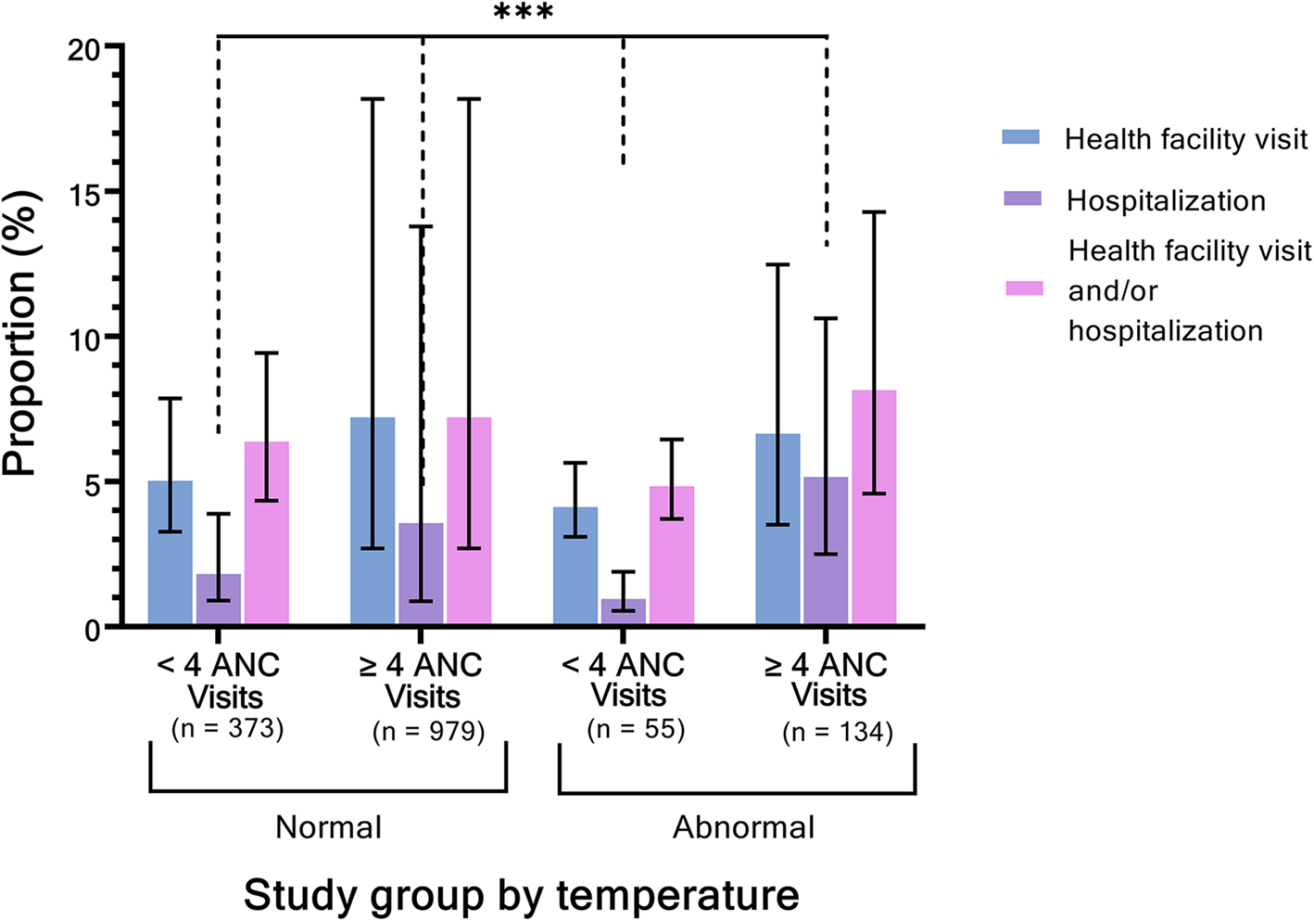
Healthcare utilization reported during the postpartum period, by ANC utilization in pregnancy and temperature during inpatient delivery stay. legend: Tests of association between healthcare utilization and study group were performed using chi-squared tests, with * indicating *P*-value < 0.1, ** indicating *P*-value < 0.05, and *** indicating *P*-value < 0.01. Error bars illustrate 95% confidence intervals. Abbreviations: ANC = antenatal care

The proportions of each group that reported having received each type of health service in the postpartum period, including visiting health facility for care, hospitalization, or general postpartum contact with healthcare (health facility visit and/or hospitalization) are illustrated in Fig. 1. The proportion who visited a health facility ranged from 4.2% (95% confidence interval [CI] 3.1 – 5.6%) for normothermic participants who attended ≥ 4 ANC visits to 7.4% (95% CI 2.7 – 18.5%) for participants with abnormal temperatures who attended < 4 ANC visits. The proportion reporting visiting a health facility and/or hospitalization ranged from 4.9% (95% CI 3.7 – 6.4%) for those normothermic participants who attended ≥ 4 ANC visits to 7.6% (95% CI 4.1 – 13.6%) for participants with abnormal temperatures who attended ≥ 4 ANC visits. The only statistically significant difference between the four groups was the proportion hospitalized, with 1.9% (95% CI 0.9 – 3.9%) of normothermic participants who attended < 4 ANC visits, 1.0% (95% CI 0.6 – 1.9%) of normothermic participants who attended ≥ 4 ANC visits, 3.7% (95% CI 0.9 – 14.0%) of participants with abnormal temperatures who attended < 4 ANC visits, and 4.5% (95% CI 2.0 – 9.8%) of participants with abnormal temperatures who attended ≥ 4 ANC visits reporting hospitalization (*P* = 0.01). Data on neonatal and perinatal outcomes are included in other research based on this cohort [23, 24].

### Regression modeling of antenatal care and abnormal temperature with healthcare utilization

In separate multivariable logistic regression models of the association between one abnormal temperature recorded or ≥ 4 ANC visits and postpartum healthcare adjusted for potential confounders including age, marital status, parity, residence in Mbarara, referral to MRRH for delivery, and HIV serostatus, neither variable was significantly associated with postpartum healthcare service utilization (Table 2). In a multivariable logistic regression model adjusted for potential confounders including both explanatory variables and an interaction term between the explanatory variables (dichotomized abnormal temperature × dichotomized ANC attendance), the interaction term was not statistically significant. Furthermore, in this model, abnormal temperature and attendance at ≥ 4 ANC visits were also not significantly associated with postpartum healthcare service utilization. Results were similar in sensitivity analyses using multivariable regression models separately examining postpartum hospitalization and healthcare facility visits, separately examining febrile and hypothermic groups with a binary temperature variable, and separately examining febrile and hypothermic groups with a continuous temperature variable.

**Table 2:** Multivariable logistic regression analysis of factors associated with postpartum health service utilization

Variable	Health facility visit and/or hospitalization aOR (95% CI)	*P*-value
**Multivariable, one explanatory variable**		
Abnormal temperature	1.09 (0.54, 2.21)	0.813
≥ 4 ANC visits	0.85 (0.51, 1.41)	0.526
**Multivariable with temperature × ANC interaction**		
Abnormal temperature × ≥ 4 ANC visits	2.28 (0.42, 12.31)	0.337
Abnormal temperature	0.59 (0.13, 2.66)	0.492
≥ 4 ANC visits	0.77 (0.45, 1.32)	0.344

All logistic regression models adjusted for participant age, marital status, parity, residence in Mbarara, referred to MRRH, and HIV serostatus

*Abbreviations*: *ANC* Antenatal care, *CI* Confidence interval, *aOR* Adjusted odds ratio, *MRRH* Mbarara Regional Referral Hospital, *HIV* Human immunodeficiency virus

## Discussion

In this cohort of 1,538 semi-rural dwelling Ugandan women, we found that contact with healthcare during the postpartum period was very low, with only 86 participants (5.6%) reporting that they visited a health facility and/or were hospitalized during the 6 weeks postpartum. PNC, self-reported by participants as visiting a health facility and/or hospitalization, varied little between each subgroup, with rates of contact ranging between 4.9 – 7.6%. Of the 86 women who reported visiting a health facility and/or hospitalization, 51.2% reported having a post-discharge infection, 43.0% reported having a post-discharge fever, and 68.6% delivered by cesarean section. Though the overall proportion who reported post-discharge health facility visit and/or hospitalization is very low, those who did had high proportions of fever, infection, and cesarean, which suggests that the post-discharge PNC may have been focused on post-cesarean care and evaluation and treatment of possible infection. None of the logistic regression models adjusted for potential confounders, including models with health facility visit and hospitalization as separate outcomes or including an interaction term between antenatal care and intrapartum fever, had a significant association between abnormal temperature during delivery or ANC attendance with PNC.

Our results differ from previous studies, which found overall positive associations between ANC and PNC in Ethiopia, Nigeria, and Uganda [19, 25–27]. However, prior studies have also demonstrated that rurality is negatively associated with PNC in sub-Saharan Africa [11, 21]. Since 652 (44.7%) of our participants do not reside in Mbarara district, indicating they likely reside in rural settings, this could partially explain differences between our results and prior studies. While this this is a comparable to the rates of rurality in similar studies, these studies also reported high numbers of participants living in proximity to health facilities [26, 27]. A qualitative study of mothers in rural Kenya notes that postpartum recovery was a common barrier to seeking PNC [28]. It is possible that a combination of factors, including the high proportion of cohort participants who are rural dwelling, combined with a high cesarean delivery rate leading to prolonged postpartum recovery, led to the low rates of postpartum contact with healthcare observed in this study.

The main strength of this study is the systematic documentation of participants’ hospitalization for delivery, including routine vital signs measurement indicating low risk of misclassification by post-delivery temperature measurement. Additionally, 99% of all eligible women screened agreed to be enrolled into the primary study indicating low risk of selection bias. The study location is also a strength to the study. We carried out the study at MRRH to leverage existing research infrastructure, and selected this site because its semi-rural setting allows our results to be more generalizable results to wider range of sub-Saharan African settings. Our study also has several limitations. Firstly, we asked participants about PNC using a general question about number and timing of visits to health care facilities, which may not capture all PNC. The very low proportion reporting PNC and high proportion of fever and infection among those who did report receiving PNC suggests that some participants may not have reported routine PNC visits and only reported problem-focused visits. However, the overall low proportion reporting visiting a health facility and/or hospitalization likely does indicate low PNC overall. Another limitation is the low number of participants in the febrile subgroups, which limited power to detect a modifying relationship of abnormal temperature on the association between ANC attendance and PNC. Though the interaction term in our model was not significant, this could be due to small sample size in these subgroups. Further research with a larger sample and more explicit and disaggregated interview instruments is needed to better explore the factors related PNC in rural and semi-rural populations living in resource-limited environments. Furthermore, the modifying relationship of other intrapartum factors including severe maternal illness during delivery, should also be explored in larger cohort studies. Lastly, while we evaluated the relationship between ANC and PNC and effect of abnormal maternal temperature on this relationship, we did not assess the quality of the ANC receive.

The WHO recently increased the recommended number of ANC visits four to eight, which adds to the urgency to better understand ANC and PNC quality in resource-limited settings, and factors influencing both quality of care and attendance [7]. The quality of the services provided during ANC and the recommendations made are crucial determinants of retaining mothers in the continuum of perinatal care and improving maternal and child morbidity and mortality. Future work should evaluate the impact of PNC quality and the marginal return of each ANC visit in terms of retention within the cascade of perinatal care and maternal and child outcomes, especially in the context of the increased number of ANC visits recommended by the WHO.

## Conclusion

We found very low rates of post-discharge PNC access in Uganda after hospital-based delivery. These results reinforce the need to promote the development and implementation of perinatal service models that account for the individual’s geographic and sociodemographic context in order to promote good health practices for mothers in diverse settings.

## Data Availability

The datasets generated during and/or analyzed during the current study are available via contacting the corresponding author on reasonable request.

## Abbreviations

ANC: Antenatal care
CI: Confidence interval
MRRH: Mbarara Regional Referral Hospital
PNC: Postnatal care
WHO: World Health Organization
