# Correction: The interaction between antenatal care and abnormal temperature during delivery and its relationship with postpartum care: a prospective study of 1,538 women in semi-rural Uganda

**DOI:** 10.1186/s12884-022-05293-8

**Published:** 2022-12-21

**Authors:** Nicholas E. Rahim, Joseph Ngonzi, Adeline A. Boatin, Ingrid V. Bassett, Mark J. Siedner, Godfrey R. Mugyenyi, Lisa M. Bebell

**Affiliations:** 1 Medical Practice Evaluation Center, Massachusetts General Hospital, Boston, USA; 2 Department of Obstetrics and Gynaecology, Faculty of Medicine, Mbarara University of Science and Technology, Mbarara, Uganda; 3 Department of Obstetrics and Gynecology and Center for Global Health, Massachusetts General Hospital, Boston, USA; 4 Department of Medicine, Division of Infectious Diseases, Medical Practice Evaluation Center, Massachusetts General Hospital, Harvard Medical School, Boston, USA; 5 Department of Medicine, Division of Infectious Diseases, Medical Practice Evaluation Center, Center for Global Health, Massachusetts General Hospital, Harvard Medical School, Boston, USA; 6 Mbarara University of Science and Technology, Mbarara, Uganda; 7 Department of Medicine, Division of Infectious Diseases, Medical Practice Evaluation Center, Center for Global Health, Massachusetts General Hospital, Harvard Medical School, 55 Fruit St, GRJ-504, Boston, MA 02114 USA

**Correction: BMC Pregnancy Childbirth 22, 860 (2022)**

**https://doi.org/10.1186/s12884-022-05207-8**

Following publication of the original article [1], the authors identified an error in Fig. 1. The correct figure is given below.

**Fig. 1:**
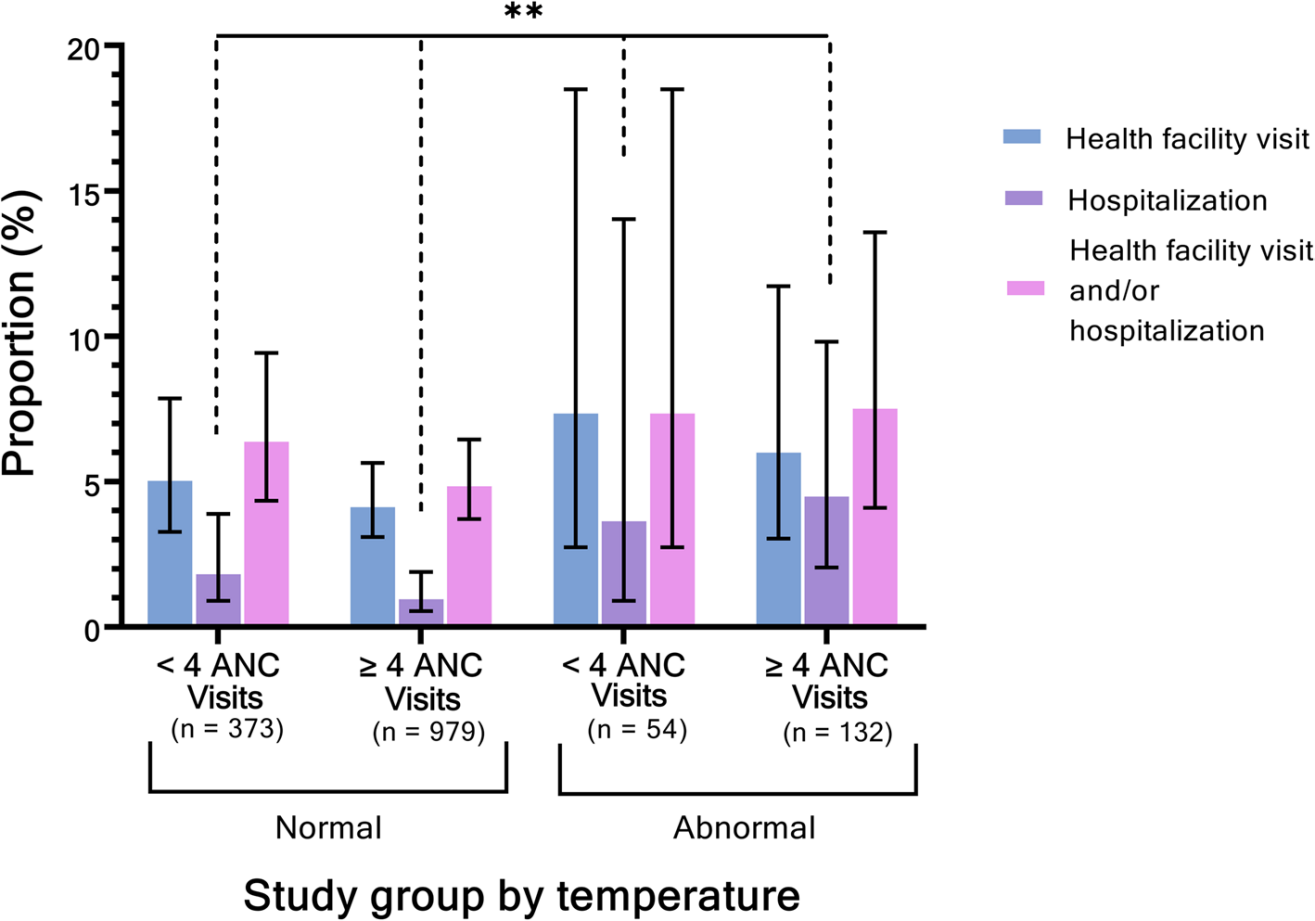
Healthcare utilization reported during the postpartum period, by ANC utilization in pregnancy and temperature during inpatient delivery stay. legend: Tests of association between healthcare utilization and study group were performed using chi-squared tests, with * indicating *P*-value < 0.1, ** indicating *P*-value < 0.05, and *** indicating *P*-value < 0.01. Error bars illustrate 95% confidence intervals. Abbreviations: ANC = antenatal care

The original article [1] has been corrected.
